# An Aberrant Seminoma as a Complication in an Undescended Testis: A Case Report and Review of Literature

**DOI:** 10.7759/cureus.39717

**Published:** 2023-05-30

**Authors:** Deepali Trimukhe, Suresh Phatak, Avinash Dhok, Kajal Mitra, Suchita Bahurupe

**Affiliations:** 1 Radiodiagnosis, N. K. P. Salve Institute of Medical Sciences & Research Centre and Lata Mangeshkar Hospital, Nagpur, IND

**Keywords:** cryptorchidism, undescended testis, non-seminomatous germ cell tumor, seminomatous germ cell tumor, testicular germ cell tumor, testicular tumor

## Abstract

Testicular germ cell tumors are testicular neoplasms in young and middle-aged men. Undescended testis dramatically increases the risk of testicular germ cell tumors. We report the case of a 33-year-old male who complained of swelling and pain in his lower abdomen. The patient also had an undescended left testis. An intrabdominal mass was detected on ultrasound that was further characterized using contrast-enhanced CT. Imaging findings suggested testicular germ cell tumor, developing as a complication in the undescended testis. The patient was operated and the diagnosis was confirmed on histopathological examination.

## Introduction

The most frequent testicular neoplasm is the testicular germ cell tumor. The median age for diagnosis of seminoma is typically between 35 to 39 years. Compared to non-seminomatous germ cell tumors (NSGCTs), patients with seminoma are usually diagnosed when they are about 10 years old on average [1]. Conditions like genitourinary anomalies and cryptorchidism are prevalent in male infants, affecting up to 30% of premature male infants and 15 to 3% of full-term male infants. If left untreated, cryptorchidism can raise the likelihood of infertility and testicular cancer [2]. Individuals who had germ cell tumors previously have a higher risk of second testicular malignancy, with the incidence around 2% to 3% [1].

We report the case of a 33-year-old male with a palpable painful mass in the left hypogastric region, who also had undescended testis on the left side, diagnosed subsequently as seminoma on post-surgical histopathological examination.

## Case presentation

Patient information

A 33-year-old male presented with chief complaints of progressive swelling and pain in the lower abdomen for one month and more on the left side. The patient also had a history of undescended left testis.

Clinical assessment

A palpable mass was noted in the hypogastric region in the left paramedian location. The mass was a little tender on palpation. The left scrotal sac was empty. The right testis and epididymis were normal in the right scrotal sac. 

Imaging assessment

**Figure 1:**
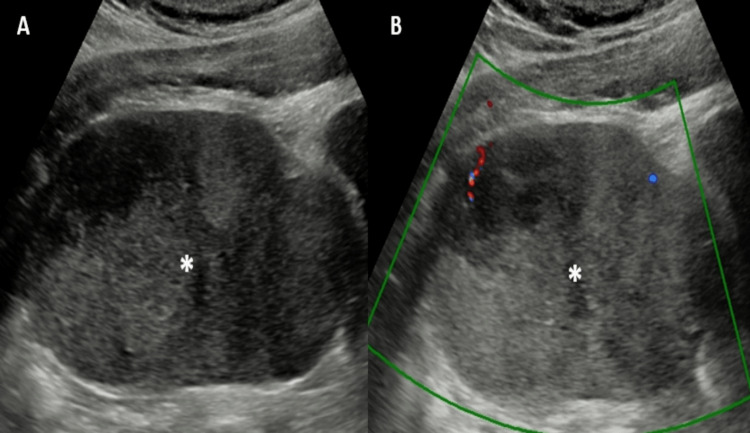
Ultrasound and color Doppler of the abdomen A: Ultrasound shows a well-defined iso-hyperechoic solid mass (asterisk) in the left hypogastric region. Multiple anechoic areas within the mass represent necrotic changes. B: On color Doppler, the mass shows minimal peripheral vascularity.

Ultrasound showed a well-defined iso-hyperechoic solid mass of size 75 x 67 x 66 mm in the hypogastric region on the left side. Multiple hypo-anechoic areas suggesting necrosis were also present within the mass. No calcification was present within the mass. On color Doppler, the lesion showed minimal peripheral vascularity (Figure 1).

The rest of the abdominal organs were normal. On scrotal ultrasound, the left testis was not visualized in the left scrotal sac. Right testis and epididymis were normal. 

**Figure 2:**
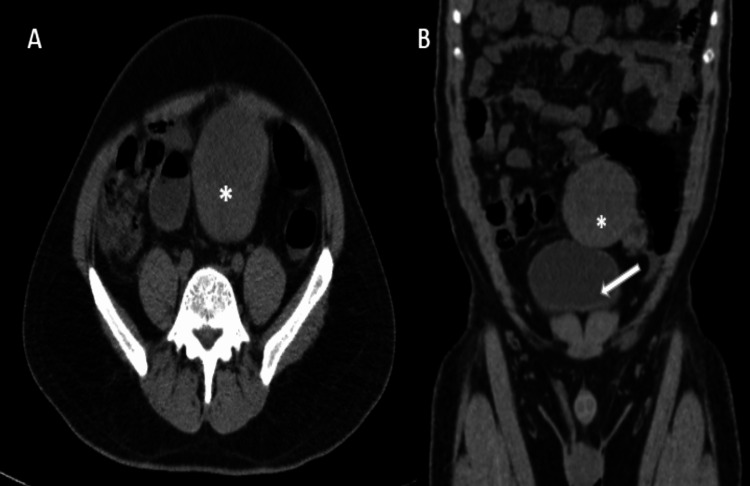
Axial (A) and coronal (B) section of plain CT abdomen showing well-defined lobulated solid soft tissue attenuation mass (asterisk) in the left hypogastric region in the supra-vesical area (urinary bladder marked by white arrow)

A CT scan showed a well-defined lobulated solid soft tissue attenuation (HU +15 to +32) mass of size 75 x 67 x 66 mm in the hypogastric region on the left side in the supra-vesical area (Figure 2).

**Figure 3:**
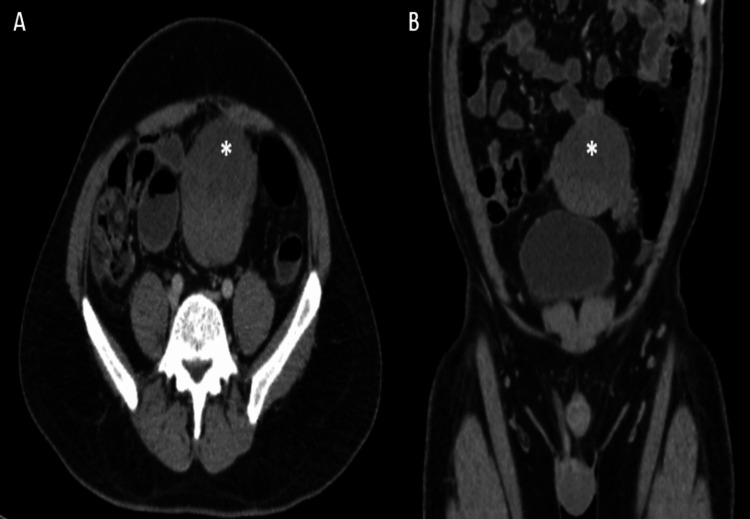
Axial (A) and coronal (B) sections of post-contrast CT show heterogeneous post-contrast enhancement of mass and non-enhancing necrotic components (asterisk)

In a post-contrast study, the mass showed heterogeneous enhancement with a non-enhancing necrotic area (Figure 3).

The left testis was not visualized in the left scrotal sac in view of the history of undescended testis. The right testis was normal. No evidence of any abdominal lymphadenopathy was noted. A diagnosis of a testicular tumor developing as a complication of long-standing undescended testis was made. Subsequently, the patient underwent surgery and resection of the mass was performed.

Histopathology

On the gross cut specimen, the mass revealed a well-circumscribed nodular neoplasm. On microscopy, the nodules were comprised of sheets of polygonal cells with well-defined cell borders, large squares of nuclei, vesicular chromatin, prominent eosinophilic nucleoli, and a moderate amount of vacuolated cytoplasm. The nodules were separated by thin fibrous bands. The stroma showed interspersed perivascular aggregates of lymphocytes. On immunohistochemistry, tumor cells expressed SALL-4, OCT-4, and CD-117. Epididymis and vas deferens seen in the cut section were uninvolved by the tumor. These findings were consistent with classical seminoma.

Follow up

The postoperative period was uneventful and the patient recovered well. After discharge, the patient was referred to the oncology department for further management.

## Discussion

Testicular germ cell tumor is an important testicular neoplasm. The evolution of germ cell testicular tumors is thought to start with abnormalities that occur during the development and migration of germ cells. Various factors have been identified as potential predisposing factors for testicular germ cell tumors, including a presence in the family, undescended testis, small-sized atrophic testis, hypospadias, oligospermia, testicular dysgenesis syndrome, and exposure to estrogen from external sources during the antenatal period. Cowden syndrome, a hereditary condition, has also been a risk factor for developing testicular germ cell tumors [3]. In the embryo, at the stage of the bilaminar disk, gonocytes, or primitive germ cells, can be determined at the age of two weeks of gestation. These cells possess stem cell-like markers, including octamer binding transcription factors 3 and 4 (OCT3/4) and c-KIT receptor tyrosine kinase. Following extensive migration, primitive germ cells occupy the parenchyma of the testis and convert into mature sex cell progenitors during puberty. As part of this process, they relinquish their primitive markers. However, there is a theoretical risk that primitive germ cells may be deposited along the migration pathway during this process, potentially leading to arrested primitive germ cell development and subsequent germ cell neoplasm in situ (GCNIS) proliferation. Pubertal activation of the hypothalamic-pituitary-gonadal axis can trigger this proliferation, which may result in the occurrence of extragonadal germ cell tumors [3]. The risk of germ cell tumors increases by five to 10 times in cases of undescended testis [4]. Our patient, too, had an undescended testis.

In 2016, the classification for germ cell tumors was revised by the World Health Organization (WHO) to include a new term for the precursor lesion: germ cell neoplasm in situ (GCNIS). The tumors are divided into two groups depending on whether chromosome 12p amplification is present or not: GCNIS-related tumors exhibit chromosome 12p amplification; non-GCNIS-related tumors do not exhibit chromosome 12p amplification.

Tumors related to GCNIS comprise seminomas, choriocarcinomas, embryonal carcinomas, teratomas, post-pubertal yolk sac tumors, NSGCTs, and "burned-out" testicular germ cell tumors. Non-GCNIS-related tumors include spermatocytic tumors, prepubertal teratomas, and yolk sac tumors [3].

Ultrasound of seminomas usually shows a homogenous or low-echogenic mass. Small, well-defined nodules or a large mass that replaces the entire testis are both possible presentations. Smaller seminomas are typically homogenous and solid; larger tumors may be more heterogeneous in echotexture with calcification and cystic changes. On a color Doppler study, they usually show vascularity, but this may not be seen in cases of smaller tumors. Non-seminomatous germ cell tumors usually show irregular borders and a combination of low and high echogenicity on ultrasound. These tumors frequently contain calcifications and cystic elements, which have been detected in as many as 40% of cases [4].

Computed tomography is the preferred modality for evaluating retroperitoneal lymph nodes. The most common site for testicular cancer to metastasize is the retroperitoneal lymph nodes due to their lymphatic and venous drainage pathways. According to the National Comprehensive Cancer Network (NCCN), if abdominal CT shows retroperitoneal metastasis in a seminoma, a CT chest should be performed to assess thoracic lymphadenopathy and pulmonary metastasis. Chest radiographs may be done for staging in cases where there are no retroperitoneal lymph nodes on CT [5].

An MRI offers the advantage of evaluating tumors based on their T1 and T2 features, enabling the distinction of soft tissue, fat, and fluid. Testicular seminoma typically shows a homogeneously isointense signal on T1 and a hypointense signal on T2 weighted imaging (WI), and they exhibit enhancement of fibrovascular septa more than the surrounding tumor tissue on post-contrast T1WI. In contrast, more heterogeneity is shown by NSGCTs, with iso-hyperintensity on T1WI and T2WI. An MRI is the preferred modality for brain imaging as part of the initial staging in special clinical situations, e.g., in choriocarcinoma, where there are high chances of brain metastasis [5].

Positron emission tomography is frequently recommended because it has been demonstrated to be more sensitive than CT while maintaining a comparable level of specificity. A PET's usefulness in initial staging is limited if the metastatic disease has already been identified [6].

## Conclusions

Testicular cancer in an undescended testis is the most common but critical complication of cryptorchidism. Regular follow-up and surveillance are mandatory to detect testicular tumors early and ensure prompt treatment. Ultrasound, CT, and MRI are extremely important modalities in the diagnosis and management of testicular tumors. 
